# Transactions

**Published:** 1883-11-20

**Authors:** 


### TRANSACTIONS

Of the Medical Society of the State of West Virginia, sixteenth annual session, held in Grafton, May 16th and 17th, 1883. Instituted April 10th, 1867.

A book of 83 pages, neatly published, and kindly sent us by the Secretary, Dr. S. L. Jepson, of Wheeling, Virginia. The roll of membership numbers 140 names. The work contains the minutes and the following papers of interest:

Annual Address ot the President, Dr. B. W. Allen, of Morgantown.

Report on Epidemic Diseases--By R. W. Hall, M. D., of Mannington.

Report on New Remedies--By J. M. Lazzell, M. D., of Fairmount.

The Germ Theory of Disease--By E. C. Meyers, M. D., of Wheeling.

Abuse of Ergot in Obstetric Practice--By D. Porter, M. D., of Clarksburg.

Insanity as a Disease--By George H. Carpenter, M. D., of Moorefield.

Puerperal Fever–By S. L. Jepson, M. D., of Wheeling.

Case of Intra-Peritoneal Hæmatocele--By R. W. Hall, M. D., of Mannington.

---

### TRANSACTIONS.

Transactions of the Texas State Medical Association, fifteenth annual session, held at Tyler, Texas, April 24th, 25th, 26th and 27th, 1883.

For the above we are indebted to the kindness of W. J. Burt, M. D., Austin, Texas, Secretary of the Association. It is a neatly gotten up book of 315 octavo pages, and contains, in addition to the Minutes, Constitution, Roll of members, etc , the following interesting papers:

Address of the President—S. F. Starley, M. D.
Hæmaturi Miasmatica—Sam. R. Burroughs, M. D.
Chromic Acid in Uterine Hemorrhage—W. F. Starley, M. D.
Embryo Physician as a Specialist—T. H. Nott, M. D.
Mineral Waters of Texas--J. M. Willis, M. D.
Chloroform in Obstetric Practice—H. C. Ghent, M. D.
Management of Placental Membranes—Alex. W. Acheson M. D,
Cæsarian Section--Post-mortem—J. J. Burroughs, M. D.
Summer Complaints in Children--S. W. Johnson, M. D.
Puerperal Convulsions—J. L. Cunningham, M D.
Decadence of the Family and Forced Abortion, etc.—W. J. Burt, M. D.
Resume of Surgery—A. P. Brown, M. D.
Ulcer and Intussusception, etc.—J. D. Osborne, M. D.
Antiseptic Surgery–J M. Fort M. D.
Difficulties of Tracheotomy—T. H. Nott, M. D.
Treatment of Internal Hemorrhoids—Will. B. Davis, M. D.
Sponge Grafting—Drs. Beall and Adams.
Treatment of Ulcers, etc.—Wm. Penny, M. D.
Laceration, Cervix, Perineum, and Cancer, etc.—T. J. Wagley, M. D.
Hemorrhoidal Tumors-Meatus Urinarious—Arthur T. Wolf, M. D.
Ovariotomy–J. J. Burroughs, M. D.
Ovariotomy—S. F. Starley, M. D.
Ovariotomy—T. D. Wooton, M. D.
Uterine Polypi, etc.—J. C. B. Renfro, M. D
Congenital Absence of the Uterus, etc.—M. J. Birdsong, M. D.
Vesico-Vaginal Fistula—Hilliary Ryan, M. D.
Progress of Ophthalmology—George P. Hall, M. D.
Cataract Operations—R. H. Chilton, M. D.

